# Social acceptance of livestock-administered endectocides for malaria control in Vhembe District, Limpopo Province, South Africa

**DOI:** 10.1186/s12936-022-04334-z

**Published:** 2022-10-28

**Authors:** Takalani I. Makhanthisa, Leo Braack, Maria S. Bornman, Heike Lutermann

**Affiliations:** 1 Department of Zoology & Entomology, Mammal Research Institute, University of Pretoria, Pretoria, South Africa; 2 Faculty of Health Sciences, UP Institute for Sustainable Malaria Control, University of Pretoria, Pretoria, South Africa; 3 Faculty of Tropical Medicine, Malaria Consortium, Mahidol University, Bangkok, Thailand

**Keywords:** Community engagements, Mosquitoes, Insecticides, Vector control

## Abstract

**Background:**

Malaria continues to be a leading cause of morbidity and mortality in Africa and conventional malaria control strategies, such as indoor residual spraying and insecticide-treated bed nets, have limited effectiveness for some malarial vectors. Consequently, the development of alternative or supplementary strategies is required. One potential strategy is the use of livestock-administered endectocides to control vector mosquitoes that feed outdoors on livestock. However, since this strategy requires support from local communities and livestock owners consenting for their animals to be treated, it can only be implemented if agreed to by affected communities. The aim of this study was to assess the social acceptance of the use of livestock-administered endectocides in the malaria endemic villages of Vhembe District, Limpopo Province, South Africa, where malaria incidence is high.

**Methods:**

Questionnaires were administered to 103 livestock-owning households from four villages, namely, Gumbu, Malale, Manenzhe and Bale. The assessment included questions on the acceptability of the strategy, the type and number of livestock owned, distances between houses and kraals (overnight pens) as well as previous use and awareness of endectocides. The results were analysed using descriptive statistics and multinomial logistic regression.

**Results:**

The types of livestock owned by the participants comprised, cattle, goats, sheep and donkeys, with the most dominant being goats (n = 1040) and cattle (n = 964). The majority of kraals were less than 10 m from homesteads. Most participants (72.5%) were already using chemicals to treat their livestock for parasites. All participants were amenable to the implementation of the strategy, and would give consent for their animals to be treated by endectocides.

**Conclusions:**

The use of livestock-administered endectocides appears to be a feasible and acceptable approach for control of animal-feeding malaria vector species in the malaria endemic villages of Vhembe District. This is based on a high percentage of rural residents keeping suitable livestock close to their homes and expressing willingness to use endectocides for mosquito control.

## Background

Malaria is a major global public health threat that continues to impact the well-being of people and nations. The 2021 Malaria Report of the World Health Organization (WHO) states that in 2020, there were 241 million cases associated with 627,000 deaths globally [1]. This increase of 14 million cases compared to the 229 million cases that were reported in 2019 [1] occurred one year after commencement of the COVID-19 pandemic and the resulting service disruptions [2]. Most of the cases (95%) were from the WHO African region followed by WHO Eastern Mediterranean and South-East Asian region, which had 2.4% and 2% cases, respectively [1].

Malaria is caused by *Plasmodium* parasites, the most lethal and common in Africa being *Plasmodium falciparum* [3]. Female *Anopheles* mosquitoes of a particular range of species are responsible for transmitting *Plasmodium* parasites to human hosts [4]. Major malaria vectors in Africa are *Anopheles arabiensis*, *Anopheles gambiae* and *Anopheles funestus* [5]. Several other *Anopheles* species are primary vectors in more limited geographic areas, while some species transmit malaria as secondary vectors [6]. *Anopheles arabiensis* is the primary malaria vector in South Africa [7]. It is zoophilic, preferring to feed on cattle but will also readily feed on humans, mostly outdoors where they prefer to rest [8]. These attributes make it much more difficult to target *An. arabiensis* using conventional vector control tools such as insecticide-treated bed nets (ITNs) and indoor residual spraying (IRS).

In South Africa, malaria transmission is seasonal and most intense in the warm rainy season, which is typically from September to May [9]. There are three malaria endemic provinces, Limpopo, KwaZulu-Natal and Mpumalanga [9]. Vhembe District of the Limpopo Province has the highest malaria incidence and prevalence in the country [10]. The IRS coverage in the malaria-affected areas of Limpopo Province was between 85 and 90% over the period 2010 to 2014 [11]. Despite annual implementation of IRS, the Vhembe District remains heavily burdened with malaria [10], strongly suggesting that suitable supplementary or new control methods should be identified for deployment.

Vhembe District is in the far north of South Africa and borders with Zimbabwe, Mozambique and Botswana [9]. Mozambique and Zimbabwe are high-transmission malaria-endemic countries, which contributes to the number of cases due to imported malaria [10], such cases setting up local foci for mosquito infection. Vhembe is one of the least developed districts in South Africa with the greatest proportion of rural inhabitants [12] and it is estimated that over half its population lives in poverty [13]. Malaria is recognized as a disease of poverty and is concentrated in poor areas [14]. Poor housing structures without screened windows and doors in malaria-endemic villages increases contact between humans and mosquitoes, leading to an increase in malaria transmission [14].

Endectocides, such as ivermectin and fipronil, are antiparasitic drugs that are active against both ecto- and endoparasites in humans and animals [15]. Several studies have investigated the effect of endectocides against various malaria vectors, such as *An. arabiensis*, *Anopheles coluzzii* and *An. gambiae* in livestock, cattle in particular, and showed significant decreases in the survival and fecundity of these mosquitoes [16–18]. Although the use of livestock-administered endectocides in the Vhembe District could be beneficial in malaria control programmes, it is unclear how such a strategy would be perceived by the local communities. Before the implementation of any public health strategy, it is important to consider the community perception, attitude and knowledge towards such a strategy as acceptance can have significant impacts in its effectiveness [19]. For instance, in the Ebola outbreak that occurred in the West Africa in 2014, the local community played a key role in reducing the transmission, implementing community-based interventions including reducing body to body contact, wearing of protective gear and avoidance of crowding [20]. Another example of how the community’s acceptance is linked with the success of a health control strategy would be the patient separation from families and communities that was implemented to control the Marburg filovirus haemorrhagic fever outbreak that occurred in Angola in 2005 [21].

The willingness of local farmers to enrol their livestock in an endectocide administration malaria control scheme would be similarly crucial for any planned intervention using endectocides. As chemical intervention strategies also come at a cost, effective interventions need to consider a number of factors for targeted applications. These include not only treating a sufficient number of livestock [15] but also preferentially treating those which are closer to human habitation and are thus, most likely to attract blood-seeking mosquitoes that may then feed on human hosts [22]. Consequently, livestock in close proximity of the houses and kraals (animal pens) should preferentially be targeted for interventions.

The aim of this study was to assess the social acceptance of potential future livestock endectocide administration as an additional tool for malaria control in the malaria endemic villages of Vhembe District. The approach captured (1) possible determinants such as the type and size of livestock, (2) the relative proximity of houses and kraals (animal enclosures), (3) contributions of previous malaria experience to the communities’ attitudes towards the proposed strategy and (4) the nature and extent of already used chemicals to treat livestock.

## Methods

### Study area

**Fig. 1:**
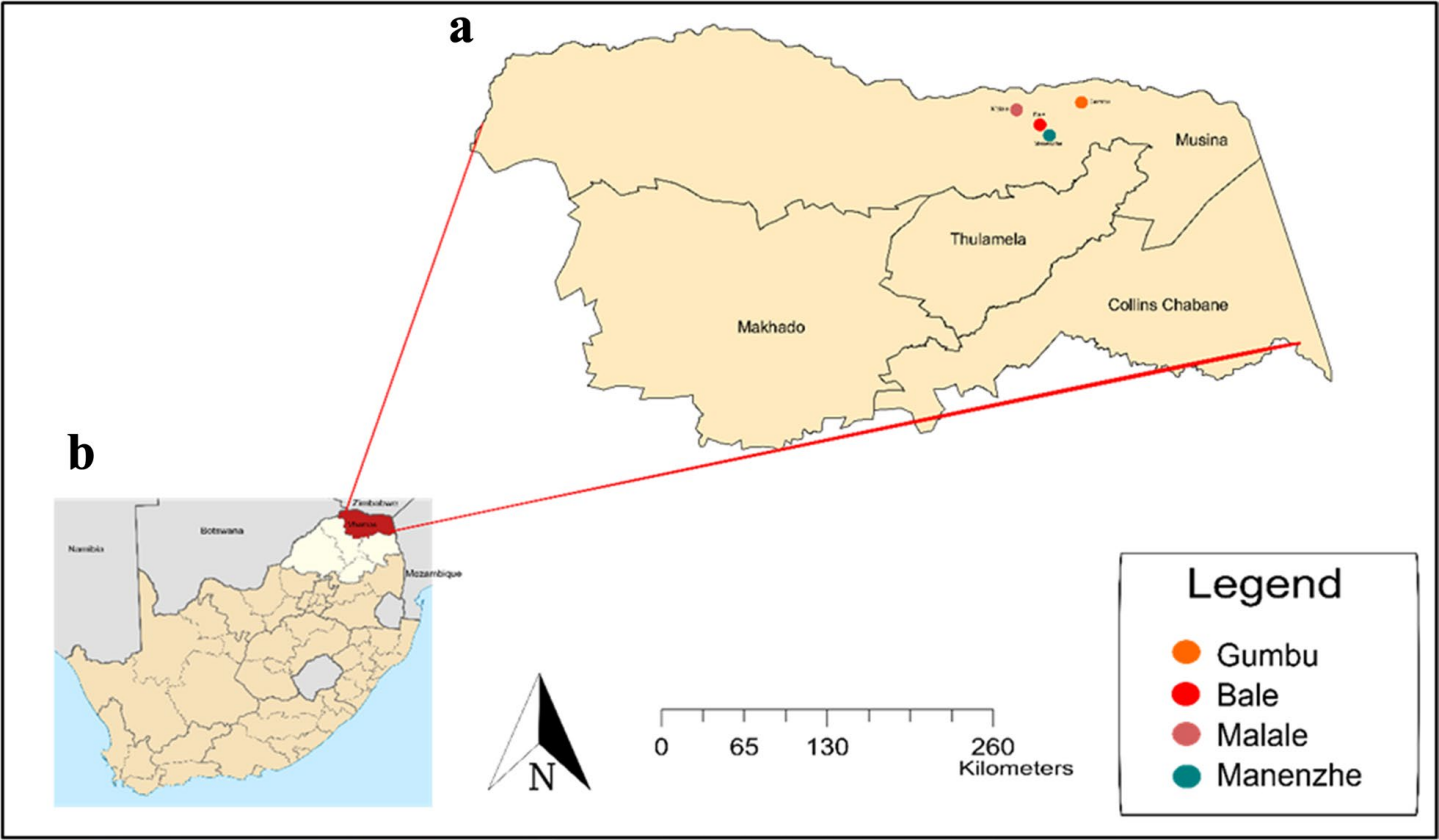
Location of the study area. **a** The four villages, Gumbu, Bale, Malale and Manenzhe are located in the Musina Municipality of the **b** Vhembe District (shown in red) of the northern Limpopo Province (shown in white). **b** Source: Htonl, https://creativecommons.org/licenses/by-sa/4.0/

This study was conducted in the Vhembe District (Fig. 1) of Limpopo Province, a 25,597 km^2^ [9] area located around 22° 85ʹ latitude and 30° 71ʹ longitude [23]. The study included four malaria-endemic villages; Gumbu, Malale, Manenzhe and Bale. Villages were chosen based on high malaria incidence and livestock population. The main activities in the Vhembe District include livestock and crop farming for subsistence purposes [23]. The district receives an average estimated annual rainfall of 820 mm [24]. A large number of households in the malaria-endemic rural areas of the Vhembe District own livestock [25].

### Questionnaire format

A questionnaire (Appendix 1) with 10 closed-ended questions and 1 open-ended question was designed to assess the social acceptability and feasibility of the use of livestock-administered endectocides in malaria-endemic rural communities. It included both qualitative and quantitative questions. In order to allow estimates of the amount of endectocides required and the financial implications of an endectocide strategy in the region, the questionnaire sought to gather information about the composition and size of the livestock kept by respondents, as well as the distance they were kept from human dwellings overnight. It is also important to know the kind of livestock owned by the households to decide on which animals should be treated with the endectocides in the community. Questions on the respondents’ view on the use of endectocides, and the extent of their own use and experience with endectocide treatment of livestock for protection against parasites as well as personal experience with malaria infection were also included. For a greater understanding by illiterate participants, the questionnaire was translated into Tshivenda (the local language) and was validated by two Tshivenda speaking field workers from the malaria offices in the Vhembe District.

### Data collection

Cattle owners were the main target group as the endectocide strategy for malaria control has primarily been investigated in cattle. However, ownership of other livestock such as goats, sheep and donkeys were also noted. Interviewers selected every second household which had livestock. The aim was to interview as many livestock owners as possible with a minimum target of 30 households. In cases where the animals were away from the houses for grazing, the presence of a kraal was used to determine if a family owned livestock. As the majority of cattle owners were illiterate, interviewers read the questionnaires to the participants and recorded the answers. In houses where cattle owners were absent, an alternative adult family member who had information about the cattle was interviewed.

### Data analysis

Collected data was analysed using the Statistical Package for the Social Sciences (IBM SPSS version 25) for statistical analyses. Due to the highly skewed responses received (see “Results” section) this was largely restricted to descriptive statistics. In order to assess whether the number of livestock owned and the amount of chemicals previously used affected the preferred type of chemical application methods used by livestock owners, a multinomial regression was employed. The dependent variable was the type of application, comprising three categories: injection, topical and oral. The actual number of livestock (continuous variable) and the volume of chemicals (low: ≤ 4 L, medium: 5–9 L and high use: ≥ 10 L) were included as independent variables.

### Ethical statement

Ethical approval for this study was obtained from the University of Pretoria Ethics Committee (Ethics no: EC063-18, 180000035). A pre-visit meeting arranged for this study by community leaders with chiefs and livestock owners was held in each of the four villages. Permission from the chiefs and community leaders of the villages was obtained before the interviews commenced. The communities were informed about the project and support was obtained in advance. The purpose of the study and how questionnaires would be administered was explained. Livestock owners were notified that the interviewers would approach them in their households. The interviewers explained to each participant that their participation was voluntary, confidential and that they could withdraw from the study at any time without explanation. Each participant was requested to sign an informed consent form after the study was explained and they had agreed to participate. Illiterate participants were asked to put a cross next to their names that the interviewers had written for them on the consent forms. This occurred after the purpose of the study and the content of the questionnaire was explained to them in their native tongue (Tshivenda language). The community field workers acted as witnesses.

## Results

### Scale of livestock ownership

A total of 103 participants from households with livestock was interviewed. Of these, 44 were from Gumbu village, while 42, 9 and 8 were from Malale, Manenzhe and Bale villages, respectively. Cattle (n = 964) and goats (n = 1040) made up the majority of ruminant livestock owned, followed by donkeys (n = 74) and sheep (n = 64). About 69% (71/103) of participants owned cattle, and over half of the cattle owners (51%) also owned goats. Only 6% of the cattle owners also owned both goats and sheep. Two participants (2%) owned all four livestock types. The majority of farmers (68%) owned 10 or less cattle and only a small number (7%) more than 30. About 18.4% and 5.8% of the participants owned 11–20 cattle and 21–30 cattle, respectively.

**Fig. 2:**
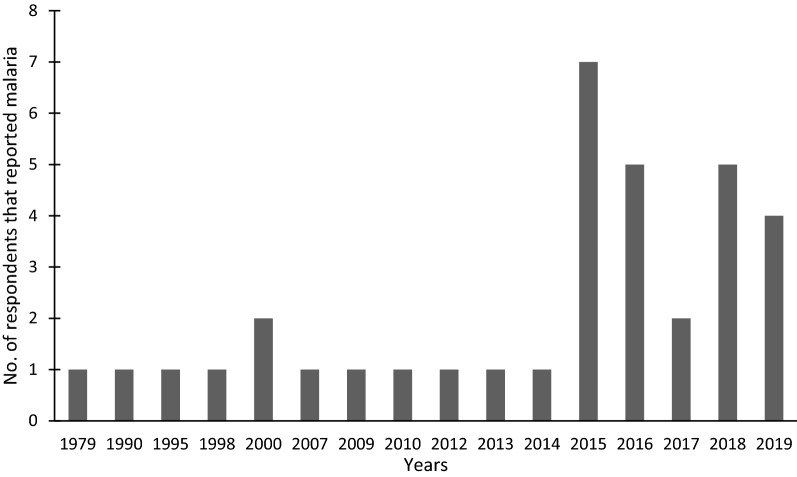
The years in which participants or their family members contracted malaria

All of the participants supported the implementation of cattle-administered endectocides and said they would be willing to let their animals be treated. The participants provided several reasons for their support of endectocides as malaria control strategy. The majority (45.6%) desired to improve the health of their animals, while 36.9% of the participants aimed to reduce malaria cases, 17.5% were concerned about both malaria and animal health. About 33.3% of the participants reported that they themselves or their family members have had malaria. Among these participants, the vast majority (85.7%) also expressed their wish to reduce malaria cases in their rationale for accepting the strategy. Based on memory, participants reported the year they or their family members contracted malaria, ranging from 1979 to the interview date. There was an apparent increase in cases from 2015 onwards (Fig. 2). When asked if they thought malaria was a problem in their communities, only few participants (6.7%) responded with “no”. These participants or their family members did not have any history of contracting malaria. The rest of the participants were aware of the malaria burden and those that have not contracted it themselves reported that they knew people that have had malaria.

The majority of livestock owners (63.8%) kept their animals in kraals at their homes at night generally less than 10 m from the house. Of these participants that kept their animals at home, about 51.5% had their kraals about 0–4 m from the houses while, 45.4% and 4.5% had their kraals at distances of about 5–9 m and greater than 10 m, respectively, from the houses. The remainder (36.2%) kept their animals at distant farms and only fetched them several times a month for health observations and treatment as necessary. The reasons for using distant farms were the limited water and food supplies available at their homes.

The majority of the participants (75.2%) reported that they had already been using chemicals to treat their livestock for ticks, worms and wounds. When they did not know the names of the chemicals, the information was collected by inspection of the containers. Redline Dip®, Supona Aerosol® and Deadline® were most frequently used (Table 1). Most participants (71.4%) could not distinguish whether the chemicals they were using treated worms or ticks specifically. However, the participants that used Ami-Tick® and Tick-Tack® reported that these were specifically for treatment against ticks. All participants that used Supona Aerosol® indicated that it was for treating wounds. All participants stated that they only used the chemicals occasionally when the animals were sick or had ticks and/or worms due to financial constraints. When asked how much of each chemical they used on their animals, some participants (22.4%) indicated that it depended on presence of parasites and how often the animals got sick. Other participants (21.1%) estimated that their chemicals lasted for a period of ± 3 months, while 15.8% and 19.7% reported that theirs lasted for ± 6 months and ± 1 month respectively. Only a few participants (5.1%) reported that they continued to take their animals for community dipping while others noted that they were no longer receiving any services from these programmes. Participants’ responses for this question depended on how many animals they owned and how often they used the chemicals. For instance, a participant with 73 cattle and 21 goats reported that they purchase 20 L of dip, which lasts them for approximately 3 months. This participant indicated that they mix the dip with water and spray on the animals. A participant with 16 cattle and 20 goats reported that they buy 1 L bottles of Amitraz, Deadline and Tick-Tack which lasts them for approximately 2 months. All the participants indicated that they found the chemicals expensive. The multinomial logistic regression indicated no significant relationship between the preferred methods of chemical applications (i.e. injection, topical and oral) and the number of livestock owned, as well as amount of the chemicals used (*X*^2^ = 45.417, df = 64, *p* = 0.962).

**Table 1:** Chemicals used on livestock and the number of participants using each of them

Product name ®	Chemical class	Number of participants (%)	Target parasites	Active ingredients (%)	Method of Administration
Amitraz	Amidine	13 (12.4)	Lice, mites, ticks	Amitraz	Topical
Deadline Dip	Pyrethroid	22 (21.0)	Ticks, lice, flies	Flumethrin (1)	Topical
Supona Aerosol	Organophosphate	23 (21.9)	Maggots in wounds, ticks	Dichlorphos (0.74) Chlorfenvinphos (0.48)	Topical
Redline Dip	Pyrethroid	39 (37.1)	Ticks, lice	Flumethrin (1)	Topical
Tick-tack	Amidine	2 (1.9)	Ticks, mites	Amitraz (12.5)	Topical
Swavet	Retinoids (Vitamin A) and tocopherols (Vitamin E)	2 (1.9)	Supplement	Vitamin A & E	Oral
Ami-Tick	Acaricide	2 (1.9)	Ticks, fleas	Amitraz	Topical
Vit-B-Co	vitamin B1 (thiamine), B2 (pyridoxine), vitamin B12 (cyanocobalamine)	1 (0.95)	Supplement	Vitamin B	Oral
Triatix	Acaricide	2 (1.9)	Ticks, lice, mites	Amitraz	Topical
Terramycin	Tetracycline antibiotics	1 (0.95)	Tick-borne gall-sickness, pneumonia, footrot	Oxytetracycline	Injection
Valbazen	Anthelmintic benzimidazole	1 (0.95)	Liver flukes, tapeworms, stomach worms	Albendazole (11.36)	Oral

## Discussion

All the participants supported the recommendation of the potential malaria control strategy. The large support for the strategy will contribute to the success of the strategy if it is to be implemented in the future. Subsistence livestock farming is important in the study area [23], despite the cattle herds of most participants being small. About 68% of the participants owned 10 or less cattle and this correlates as reported in previous studies. For example, Stroebel et al. [23] reported that in 2011, almost 60% of farmers in the Vhembe District own less than 10 head of cattle. Other studies, also in the Vhembe District, reported average herd sizes of 9 in 2014 [26] and 10.3 in 2004 [27], respectively. It will be crucial to determine a minimum herd size required for an effective treatment strategy. Possible treatment of other livestock such as goats and sheep might increase the efficacy of the strategy. In the present study, goats (48.6%) and cattle (45%) were the most common livestock, while donkeys (3.4%) and sheep (3%) were the least. Although endectocides for malaria control field experiments have mostly been conducted in cattle [16–18], other host species such as pigs and sheep have also been experimented on [28] and can be successfully used in such a strategy, and the obtained data suggests that exploring the inclusion of goats may be beneficial.

Most livestock in the Vhembe District are kept very close to the houses (less than 4 m) which would be an advantage for an endectocide-based control strategy [29]. The potential strategy is most likely to have a higher impact in areas where kraals are situated closer to houses [29]. This is because the survival and fecundity of some mosquito vectors next to the houses, that would eventually enter and bite people, will be reduced when these mosquitoes feed on endectocide-treated livestock in the kraals. Combining the potential livestock-administered endectocides strategy with other malaria control strategies such as IRS, ITNs and larval source management (LSM) could also be beneficial. While IRS and ITNs are indoor malaria control strategies, LSM is an outdoor strategy, which could also assist reduce mosquito density. The LSM method involves the application of chemical or microbial insecticides, or predatory fish, to water bodies to target the immature, aquatic stages of the mosquito in order to reduce the emergence and abundance of adult vectors, or habitat and environmental modification to reduce breeding sites [30]. This method could also help reduce the number of mosquitoes that would eventually develop and use livestock as a mode of transport from rivers and surrounding areas to kraals and subsequently houses.

The large support for implementation of livestock-administered endectocides by participants from the Vhembe District, appeared to be influenced primarily by the prospect of benefits to animal health, and secondarily by the high incidence of malaria. Belonging to the poorest regions of the country means many subsistence farmers rely heavily on their livestock for income and food [23]. Most participants were pleased to know that endectocides would also treat their livestock for other parasites such as ticks and worms. Although all participants stated that they only treated their livestock occasionally, they indicated that they can barely afford the cost of the chemicals and would appreciate it if the animals can be treated at no cost. Since malaria is linked with poverty, improving livestock health can also reduce poverty [31]. The participants used various amounts of chemicals over time, depending on the livestock sizes and how frequently the livestock got sick. Most participants with larger livestock sizes indicated that they purchase large volumes of chemicals as opposed to those with fewer animals. The majority of owners indicated that they used to take their animals for community dipping but the supply of such dipping had stopped. In South Africa, community dipping programmes were conducted by the government and were aimed at controlling parasites such as ticks [32]. The discontinuation of such programmes in most areas means that livestock owners must purchase their own chemicals, which places the livestock health of those who cannot afford the chemicals at a risk. Reinstating such programmes using livestock-administered endectocides should be a cost-effective way to not only improve human, but also animal health, and combat poverty.

Most participants indicated awareness of the local malaria burden, and many had personal experience with the disease in their family. The debilitating effects this may have had on their health, but also the lack of funds for appropriate health care and deficiencies in the healthcare infrastructure in Vhembe District, make this a financial as well as a public health challenge. This may have been a strong motivator for the positive attitude towards the proposed control strategy. The majority of participants had family members previously infected with malaria mostly between 2015 and 2019. Although this may partially be attributed to better memory of more recent incidences, this period also coincides with the global malaria increase in cases reported by the World Malaria Report [33], which also calls for urgent action in the development and implementation of new strategies. The rapid increase in malaria cases in the Vhembe District from 2015 (Fig. 2) occurred even with the use of the conventional malaria control strategy, IRS. This further stresses the need for the development of other effective malaria control strategies. Based on the responses of the participants, it appears that livestock-administered endectocides for malaria control strategy would be a well-accepted strategy by the community, which bodes well for its application in the future. It has been shown repeatedly that community acceptance is crucial for the success of disease-control strategies. Studies that were conducted to assess the community acceptance of strategies implemented to break the transmission of the Ebola haemorrhagic fever highlighted the significance of acceptance of the affected communities [34]. The interventions that were implemented during the Marburg filovirus haemorrhagic fever outbreak in Angola in 2005 provides further evidence on how high community acceptance influences the efficacy of particular disease control strategies [21].

The fact that a significant number of participants already had experience with the use of chemicals for animal health may have further affected their attitudes towards the use of such chemicals for malaria control. However, there may also be risks or disadvantages associated with the use of livestock-administered endectocides as a malaria control strategy. Different endectocides have different withdrawal periods applicable to slaughtering and consumption of milk products [35]. It is critical that livestock owners are informed of the withdrawal or waiting period. During the community meetings, interviewers followed the instructions from the manufacturers for informing livestock owners of the withdrawal periods. They emphasized that earlier slaughtering could lead to drug residues present in meat and dairy products that could cause health effects in humans such as hypersensitivity reactions, allergies and reproductive disorders [36]. Since many respondents already used chemicals to treat the livestock in the area, livestock owners were aware of the concept of withdrawal period. Most of the chemicals already used by livestock owners have a withdrawal period of seven days before cattle or sheep can be slaughtered [37] but this period increases to 28 days for Terramycin® and Valbazen® [38]. While the withdrawal period for one of the proposed endectocides (ivermectin, 21 days) falls within this time frame, it is substantially longer for fipronil with 105 days [35]. The longer withdrawal periods for fipronil might influence the communities not to allow their animals participate in an endectocide-based programme. Hence, careful consideration has to be given before choosing a particular endectocide before implementation of such control.

An additional important factor concerning livestock-administered endectocides is the route of administration. The different routes of endectocides administration (injection, topical and oral) yield different degrees of effectivity and this must be considered before the strategy can be implemented [16]. Subcutaneous injection, which is the most expensive option, is more effective than topical and oral administrations [16–18]. The results showed no significant relationship between the preferred methods of chemical applications (i.e. injection, topical and oral) and the number of livestock owned, as well as the amount of chemicals used. The topical route of administration was the most commonly used, followed by the oral form and the only injectable chemical, Terramycin®, was used by a single participant. This was expected as injectable chemicals are relatively expensive [16]. Participants were aware of the different administration routes as all application types were being used in the area and this would serve as an advantage for the livestock-administered endectocide implementation for malaria control.

## Conclusion

The size of the livestock population and the farming practices, including overnight securing of such animals close to human habitation in one of the malaria hotspots (Vhembe District) of South Africa, suggest that the use of livestock-administered endectocides for malaria control would be a feasible strategy in Vhembe. Acceptance by livestock owners appears very high as a result of the awareness of benefits to both animal and human health, as well as familiarity with chemicals used to control livestock parasites. The implementation of such a strategy could thus not only reduce the malaria mosquito vector density but improve animal health and alleviate poverty. Financial constraints would constitute the largest impediment to its implementation and would require financial assistance from government. Successful implementation of this technique would contribute evidence for potential application of endectocides in many other similar settings in Africa where zoophagic malaria vectors predominates.

## Appendix 1: Questionnaire

**Table:** 

**Questionnaire** **Introduction** South Africa is struggling to get rid of malaria. Even though the Department of Health sprays huts every year, we still cannot get rid of malaria. This is partly because many of the malaria mosquitoes bite outside the huts and they also sometimes feed on cattle instead of humans. So we want to treat cattle with a medicine that is used to kill worms inside the stomach but we know the medicine also kills mosquitoes. We want to know if you would be interested in taking part in a study to find out if we can control malaria by giving cattle the medicine for worms and then we can look if the medicine is helping to reduce the mosquito population enough to reduce malaria. The medicine we want to use is used in many parts of the world, including South Africa; it is not a new medicine, it has been used for a long time, but we want to see what it does to mosquitoes, not just worms **Disadvantages of endectocides in animal use** Besides the discomfort or a little bit of pain from the injection, there are no reported disadvantages of endectocides in animals With this background on endectocides given, please answer the following questions **What will the information you provided with be used for?** The information you provide on this form will be used for a master’s degree report and publication. It is to let malaria researchers know how the community would feel about this strategy if it was to be used in malaria control programmes. Your names and surnames will however not be made known to anyone so no-one will know you were part of this study and what answers you gave **Who can answer your questions about this project?** If you have questions about your participation in this project please contact Dr Heike Lutermann on 012 4,204,627 or Prof LEO Braack on 0,123,563,087 **Please answer the following questions** 1. Do you have cattle, and if so, how many? 2. If the use of endectocides as an outdoor malaria control strategy was to be approved, would you let us treat your cattle? Yes No Maybe 3. Whether you answered yes or no, would you please indicate reasons 4. Have you or anyone in your family ever contracted malaria? Yes No 5. If you answered yes to the above question, when was it? 6. Do you keep your cattle in a kraal at night? 7. If you answered yes to the question above, how far is the kraal from your house? 8. Do you treat your cattle for worms, and if so, what do you use? 9. Do you treat your cattle for ticks, and if so, what do you use? 10. How much of the chemical do you use for treating your animals? 11. Do you think malaria is a problem? (You can be honest!)	

## Data Availability

The datasets can be made available by the corresponding author upon reasonable request.
